# Effects of Zearalenone on the Kiss1/GPR54 System and Related Genes Expression in the Hypothalamus and Pituitary Gland of Weaned Gilts

**DOI:** 10.3390/toxins18050195

**Published:** 2026-04-22

**Authors:** Zixue Yuan, Min Zhou, Yue Luan, Lei Kong, Weiren Yang, Shuzhen Jiang

**Affiliations:** 1 Key Laboratory of Efficient Utilization of Non-Grain Feed Resources (Co-Construction by Ministry and Province), Ministry of Agriculture and Rural Affairs, College of Animal Science and Technology, Shandong Agricultural University, Tai’an 271017, China; yuanzixue_1215@163.com (Z.Y.); ly13615367939@163.com (Y.L.); 2 Shandong Provincial Animal Husbandry General Station, Jinan 250109, China; kongleiisme@163.com

**Keywords:** zearalenone, hypothalamus, Kiss/GPR54, pituitary gland, weaned gilt

## Abstract

Zearalenone (ZEA) is a potent estrogenic mycotoxin known to disrupt reproductive functions, but its precise central neuroendocrine mechanisms remain unclear. This study investigated the effects of ZEA on the hypothalamic-pituitary Kiss1/GPR54 signaling pathway in weaned gilts. A total of 32 gilts were randomly assigned to four dietary treatments contained with 0, 0.15, 1.5, or 3.0 mg/kg ZEA for a 32-day feeding trial. Histopathology, immunohistochemistry, and mRNA/protein expression analyses of GPR30, Kiss1, GPR54, GnRH, and GnRHR in the hypothalamus and pituitary gland were conducted. ZEA exposure induced significant histological damage in both tissues. In the hypothalamus, Kiss1, GPR54, GnRH, and GnRHR exhibited a non-linear response, increasing at moderate doses and decreasing at 3.0 mg/kg ZEA, whereas GPR30 expression was continuously upregulated. In the pituitary gland, GnRHR showed a similar non-linear pattern. Furthermore, high-dose ZEA down-regulated pituitary Kiss1 and GPR54 while up-regulating GnRH and GPR30 expressions. In conclusion, ZEA induces reproductive neuroendocrine toxicity through a complex, dose-dependent modulation of the Kiss1/GPR54 signaling axis. The persistent upregulation of GPR30 suggests it acts as a crucial mediator in disrupting this endocrine feedback loop within the hypothalamus and pituitary gland.

## 1. Introduction

Zearalenone (ZEA), a nonsteroidal estrogenic fungal toxin produced by Fusarium species, is a widespread contaminant of feed ingredients such as corn and wheat, presenting a serious threat to global feed and food safety [1,2]. Due to its chemical structural similarity to estradiol, ZEA can competitively bind to estrogen receptors and exert estrogen-like effects [3]. Among domesticated animals, pigs are regarded as the most sensitive to ZEA [4]. Although the European Union and China have set guidance values of 0.1 and 0.15 mg/kg of ZEA in complementary and complete feed for gilts [5,6], studies showed that ZEA contamination levels may exceed 2 mg/kg [7], leading to severe reproductive disorders such as genital swelling, uterine and mammary hyperplasia, ovarian dysfunction, and infertility [8,9]. This phenomenon is primarily attributed to the substantial transformation of ZEA into its more toxic metabolite, alpha-zearalenone (α-ZOL), following hepatic metabolism in pigs, as α-ZOL exhibits a stronger affinity for estrogen receptors [10].

The intricate regulation of animal reproductive activity is orchestrated by the hypothalamic-pituitary-gonadal (HPG) axis, with the hypothalamic secretion of gonadotropin-releasing hormone (GnRH) acting as the pivotal upstream signal that initiates and sustains reproductive function [11]. The pulsatile release of GnRH stimulates the pituitary gland, leading to the synthesis and secretion of follicle-stimulating hormone (FSH) and luteinizing hormone (LH), which in turn regulate gonadal development and activity [12]. The Kiss1/GPR54 signaling axis serves as a primary modulator of GnRH neuronal activity, acting as a vital gatekeeper for both the initiation of puberty and the maintenance of reproductive cycles [13]. Mechanistically, kisspeptin (Kiss) released by neurons within the hypothalamic arcuate nucleus stimulates GPR54 receptors on GnRH neurons, thereby orchestrating the pulsatile release of GnRH [14].

Although the reproductive toxicity of ZEA, an exogenous estrogen, is well documented [15], the specific mechanisms of its action, especially its effects on crucial regulatory pathways upstream of the HPG axis remain incompletely understood. In GT1-7 mouse hypothalamic GnRH neuronal cells, ZEA has been reported to promote GnRH secretion via activation of the G protein-coupled estrogen receptor [16]. Within the hypothalamus, ZEA directly activates kisspeptin (KP) neurons to promote GnRH secretion, thereby inducing precocious puberty [17]. Existing research indicates that prepubertal ZEA exposure can prematurely activate the hypothalamic Kiss1/GPR54 signaling pathway in rats, resulting in peripheral manifestations of precocious puberty [18]. Nevertheless, significant gaps remain in understanding the effects of ZEA on the Kiss1/GPR54 signaling pathway in the hypothalamus and pituitary of pigs, particularly during critical reproductive developmental stages such as weaning. Previous work from our laboratory demonstrated that ZEA exposure in weaned gilts leads to ovarian atrophy and uterine hyperplasia, accompanied by disruptions in serum hormone levels [19,20]. These findings suggest that ZEA’s mechanism of action may be more complex than previously understood, with its disruption of the HPG axis likely originating from interference within the upstream central regulatory system rather than being limited to terminal organs. Therefore, we hypothesized that dietary ZEA exposure in weaned gilts would disrupt the hypothalamic-pituitary Kiss1/GPR54 signaling axis. To test this, the present study investigated the dose-dependent effects of ZEA on the Kiss1/GPR54 pathway and associated gene expressions in the hypothalamus and pituitary of weaned gilts.

## 2. Results

### 2.1. The Morphological Structure of the Hypothalamus and Pituitary Gland

**Figure 1:**
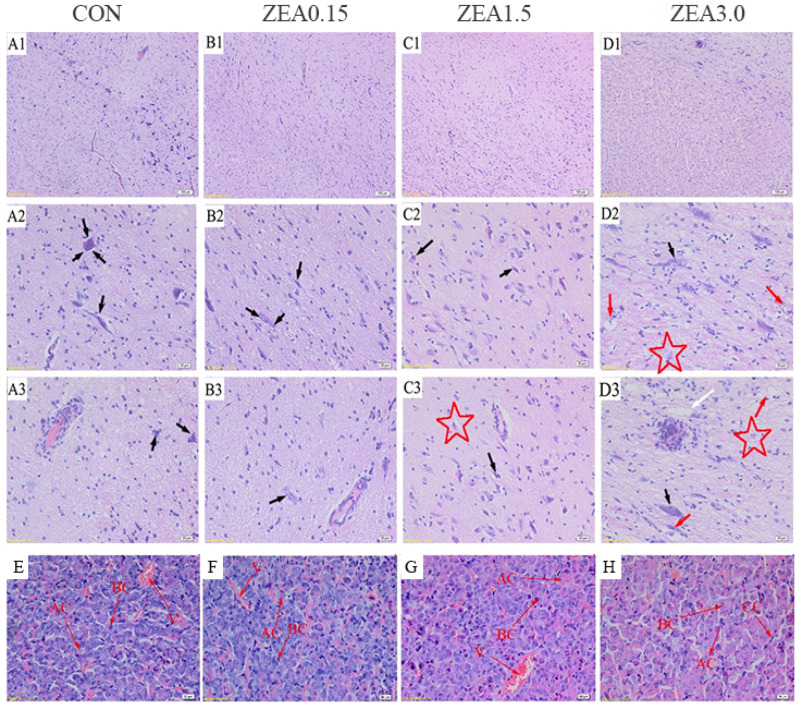
Effects of zearalenone on the histomorphology of hypothalamus (**A1**–**D3**) and pituitary gland (**E**–**H**) in weaned gilts. Tissue sections were stained with hematoxylin and eosin (**H**,**E**). Panels (**A2**,**D2**) and (**A3**–**D3**) are higher magnification views of the areas shown in (**A1**–**D1**). The treatments (CON, ZEA0.15, ZEA1.5, and ZEA3.0) received basal diets contained with 0, 0.15, 1.5, and 3.0 mg/kg ZEA, which had analyzed ZEA concentrations of 0, 0.14 ± 0.00, 1.45 ± 0.02, and 3.12 ± 0.07 mg/kg, respectively. The same as below. The red pentagrams, black arrows, white arrows, red arrows, V, AC, BC and CC represent atrophic neurons, Nissl corpuscles, cavities, ghost cells, vessels, acidophilic cells, basophilic cells and chromophobe cells, respectively. The scale bars represent 100 μm in (**A1**–**D1**) and 20 μm in (**A2**–**D2**, **A3**–**D3** and **E**–**H**).

The effects of dietary ZEA exposure at 0 (Control), 0.15 (ZEA0.15), 1.5 (ZEA1.5), and 3.0 (ZEA3.0) mg/kg on the morphology of hypothalamic and pituitary tissues were evaluated. In control (Figure 1(A1–A3)) and ZEA0.15 treatments (Figure 1(B1–B3)), neuronal and granular cells were neatly arranged, with clearly visible Nissl bodies and deeply stained nuclei. However, as dietary ZEA levels increased, the arrangement of neurons and granulosa cells became disordered, nuclear staining was lighter, and neurons atrophied (Figure 1(C1–C3,D1–D3)). Most importantly, cavities and ghost cells were observed in the ZEA3.0 group, and Nissl bodies appeared indistinct (Figure 1(D2,D3), white and red arrows).

Histomorphological analysis showed that increasing ZEA level (0, 0.15, 1.5 and 3.0 mg/kg) led to a rise in proportion of acidophilic cells and a concomitant decrease in basophils cells (Figure 1E–H). Additionally, compared with the other treatments, the cell mass boundaries indistinct and intercellular spaces were less distinct in the ZEA1.5 and 3.0 groups, resulting in a reduced size. Most notably, in the ZEA3.0 treatment, acidophilic cells exhibited hypertrophy, basophilic cells showed atrophy, and the number of chromophobe cells increased relative to all other treatments (Figure 1H).

### 2.2. Localization of GPR30, Kiss1, GPR54, GnRH and GnRHR in the Hypothalamus

**Figure 2:**
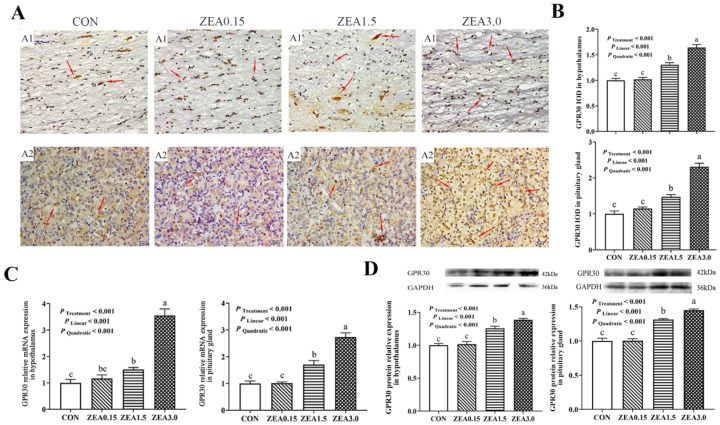
Effects of zearalenone on the GPR30 localization and expression in the hypothalamus and pituitary gland of weaned gilts. (**A**) Representative images of GPR30 immunohistochemical (IHC) staining in the hypothalamus (A1) and pituitary gland (A2); immunoreactivity is indicated by red arrows. (**B**) Quantification of GPR30 immunoreactivity, presented as Integrated Optical Density (IOD). (**C**,**D**) Relative mRNA (**C**) and protein (**D**) expression of GPR30 in hypothalamus and pituitary gland. Gilts were fed diets contained with 0 (CON), 0.15 (ZEA0.15), 1.5 (ZEA1.5), or 3.0 (ZEA3.0) mg/kg ZEA. The red arrows indicate the immunoreactivity. The scale bars represent 20 μm in (A1,A2). a, b, c Means differ significantly (*p* < 0.05).

**Figure 3:**
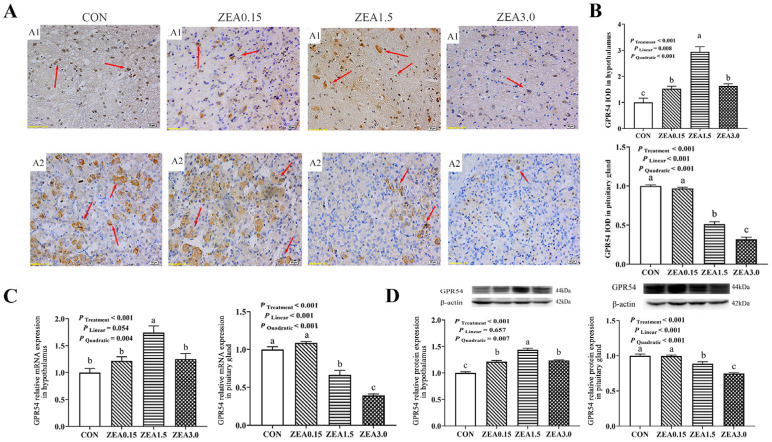
Effects of zearalenone on the GPR54 localization and expression in the hypothalamus and pituitary gland of weaned gilts. (**A**) Representative images of GPR54 immunohistochemical (IHC) staining in the hypothalamus (A1) and pituitary gland (A2); immunoreactivity is indicated by red arrows. (**B**) Quantification of GPR54 immunoreactivity, presented as Integrated Optical Density (IOD). (**C**,**D**) Relative mRNA (**C**) and protein (**D**) expression of GPR54 in hypothalamus and pituitary gland. Gilts were fed diets contained with 0 (CON), 0.15 (ZEA0.15), 1.5 (ZEA1.5), or 3.0 (ZEA3.0) mg/kg ZEA. The red arrows indicate the immunoreactivity. The scale bars represent 20 μm in (A1,A2). a, b, c Means differ significantly (*p* < 0.05).

**Figure 4:**
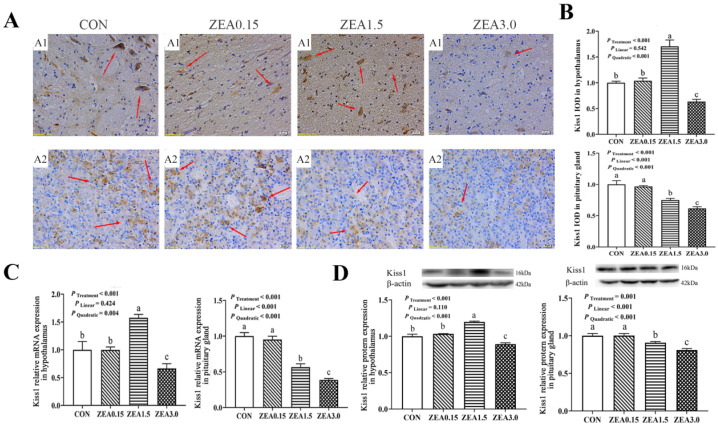
Effects of zearalenone on the Kiss1 localization and expression in the hypothalamus and pituitary gland of weaned gilts. (**A**) Representative images of Kiss1 immunohistochemical (IHC) staining in the hypothalamus (A1) and pituitary gland (A2); immunoreactivity is indicated by red arrows. (**B**) Quantification of Kiss1 immunoreactivity, presented as Integrated Optical Density (IOD). (**C**,**D**) Relative mRNA (**C**) and protein (**D**) expression of Kiss1 in hypothalamus and pituitary gland. Gilts were fed diets contained with 0 (CON), 0.15 (ZEA0.15), 1.5 (ZEA1.5), or 3.0 (ZEA3.0) mg/kg ZEA. The red arrows indicate the immunoreactivity. The scale bars represent 20 μm in (A1,A2). a, b, c Means differ significantly (*p* < 0.05).

**Figure 5:**
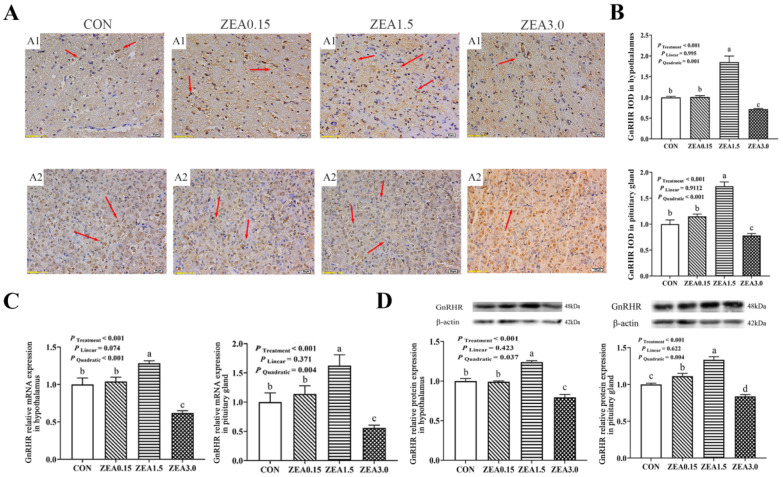
Effects of zearalenone on the GnRHR localization and expression in the hypothalamus and pituitary gland of weaned gilts. (**A**) Representative images of GnRHR immunohistochemical (IHC) staining in the hypothalamus (A1) and pituitary gland (A2); immunoreactivity is indicated by red arrows. (**B**) Quantification of GnRHR immunoreactivity, presented as Integrated Optical Density (IOD). (**C**,**D**) Relative mRNA (**C**) and protein (**D**) expression of GnRHR in hypothalamus and pituitary gland. Gilts were fed diets contained with 0 (CON), 0.15 (ZEA0.15), 1.5 (ZEA1.5), or 3.0 (ZEA3.0) mg/kg ZEA. The red arrows indicate the immunoreactivity. The scale bars represent 20 μm in (A1,A2). a, b, c Means differ significantly (*p* < 0.05).

**Figure 6:**
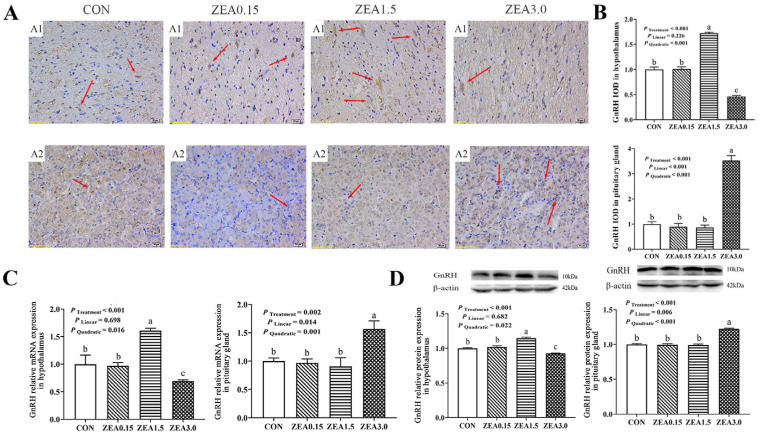
Effects of zearalenone on the GnRH localization and expression in the hypothalamus and pituitary gland of weaned gilts. (**A**) Representative images of GnRH immunohistochemical (IHC) staining in the hypothalamus (A1) and pituitary gland (A2); immunoreactivity is indicated by red arrows. (**B**) Quantification of GnRH immunoreactivity, presented as Integrated Optical Density (IOD). (**C**,**D**) Relative mRNA (**C**) and protein (**D**) expression of GnRH in hypothalamus and pituitary gland. Gilts were fed diets contained with 0 (CON), 0.15 (ZEA0.15), 1.5 (ZEA1.5), or 3.0 (ZEA3.0) mg/kg ZEA. The scale bars represent 20 μm in (A1,A2). a, b, c Means differ significantly (*p* < 0.05).

Immunohistochemical analysis revealed that GPR30 and GPR54 immunoreactivity within the arcuate nucleus (ARC) predominantly resided in the nuclear compartments of both neurons and arcuate granular cells, with minor distribution in glial cells (Figure 2(A1) and Figure 3(A1)). The IOD of GPR30 intensity increased in a dose-dependent manner (linear and quadratic effects, *p* < 0.05, Figure 2B), reaching its maximum at ZEA3.0. In contrast, Kiss1, GPR54, GnRH, and GnRHR all exhibited a significant quadratic response (*p* < 0.05, Figure 3B, Figure 4B, Figure 5B and Figure 6B), with immunoreactivity peaking at ZEA1.5 before being significantly suppressed at ZEA3.0, Kiss1, GnRH, and GnRHR even fell below the levels observed in the control group (*p* < 0.05). Kiss1 and GnRH immunoreactivities were detected mainly in the nuclei of neuronal cells, with limited presence in glial cells, furthermore GnRHR was observed primarily across all three cell types (Figure 4(A1), Figure 5(A1) and Figure 6(A1)).

### 2.3. The Relative mRNA and Protein Expression of GPR30, Kiss1, GPR54, GnRH and GnRHR in the Hypothalamus

The effects of dietary ZEA on the hypothalamic expression of key reproductive genes were dose-dependent and followed two distinct regulatory patterns. First, the expression of GPR30 showed both significant linear and quadratic increases with rising ZEA concentrations (*p* < 0.05, Figure 2C,D). This corresponded to a dose-dependent stimulation, culminating in the highest expression level in the ZEA3.0, which was significantly greater than all other treatments (*p* < 0.05). Furthermore, the stimulatory effect was already evident at ZEA1.5, which showed significantly greater expression than the control group (*p* < 0.05). Second, a significant quadratic effect was observed for the expression of Kiss1, GPR54, GnRH, and GnRHR (*p* < 0.05, Figure 3C,D, Figure 4C,D, Figure 5C,D and Figure 6C,D). Specifically, for GPR54 mRNA and protein, expression were maximally at ZEA1.5, furthermore, its protein expression in both the ZEA0.15 and ZEA3.0 were also significantly higher than in the control group (*p* < 0.05, Figure 3C,D). The relative mRNA and protein expression of Kiss1, GnRH and GnRHR were maximally induced by the ZEA1.5, reaching levels significantly higher than those in all other groups (*p* < 0.05). Conversely, increase to ZEA3.0 resulted in a significant inhibitory effect, with expression levels dropping significantly below those of the control and ZEA0.15 (*p* < 0.05, Figure 4C,D, Figure 5C,D and Figure 6C,D).

### 2.4. Localization of GPR30, Kiss1, GPR54, GnRH and GnRHR in the Pituitary Gland

Immunohistochemical staining revealed that GPR30 and GnRHR immunoreactivity was primarily localized in the nuclei of pituitary cells, with minor cytoplasmic staining observed. Conversely, Kiss1, GPR54 and GnRH were primarily distributed in the cytoplasm of pituitary cells (Figure 2(A2), Figure 3(A2), Figure 4(A2), Figure 5(A2) and Figure 6(A2)). The immunoreactivities of GPR30 and GnRH increased in a dose-dependent manner (linear and quadratic effects, *p* < 0.05, Figure 2B and Figure 6B), reaching their maximal levels at ZEA3.0, which were significantly higher than all other treatments. Conversely, Kiss1 and GPR54 were progressively downregulated by ZEA (Figure 3B and Figure 4B). Their IODs were significantly reduced at ZEA1.5 and were further suppressed at ZEA3.0 compared to controls (*p* < 0.05). GnRHR exhibited a significant quadratic effect (*p* < 0.05, Figure 5B), where its expression peaked significantly at the ZEA1.5 before being markedly suppressed at ZEA3.0 (*p* < 0.05).

### 2.5. The Relative mRNA and Protein Expression of GPR30, Kiss1, GPR54, GnRH and GnRHR in the Pituitary Gland

The relative mRNA and protein expression of GPR30 and GnRH in the pituitary gland of weaned gilts demonstrated a progressive, dose-dependent upregulation, with expression culminating at the highest ZEA dose (3.0 mg/kg), which was significantly greater than all other groups (*p* < 0.05, Figure 2C,D and Figure 6C,D). Conversely, the expression of Kiss1 and GPR54 was significantly reduced at ZEA1.5 and even more so at ZEA3.0 (Figure 3C,D and Figure 4C,D). GnRHR exhibited a significant quadratic effect (*p* < 0.05). Its expression was maximally induced at ZEA1.5 but was then strongly suppressed at ZEA3.0 to levels significantly below all other treatments (*p* < 0.05, Figure 5C,D).

## 3. Discussion

### 3.1. Histological Effects of ZEA on the Hypothalamus and Pituitary Gland of Weaning Gilts

The ZEA doses selected for this study (0.15, 1.5, and 3.0 mg/kg) warrant discussion in the context of regulatory standards. The lowest dose (0.15 mg/kg) aligns with the maximum limit permitted in China for piglet feed [6], although it slightly exceeds the stricter European Commission guidance value of 0.1 mg/kg [5]. Importantly, this concentration reflects a realistic contamination scenario, as global surveys frequently report ZEA levels in swine feed that surpass these regulatory thresholds [7]. Furthermore, the inclusion of higher doses (1.5 and 3.0 mg/kg) was indispensable for elucidating the full spectrum of ZEA’s effects over a 32-day exposure period. This dose range was crucial for uncovering the non-linear, biphasic response of the central Kiss1/GPR54 signaling axis, which transitions from initial stimulation to profound inhibition.

Building on our previous findings that ZEA causes ovarian atrophy and uterine proliferation, accompanied by disorder of serum hormone in weaned gilts [19,20], this study aimed to elucidate the underlying central regulatory mechanisms. As a first step, we performed a histological evaluation of the hypothalamic–pituitary axis. Neurons, as the basic structural and functional units of the nervous system, rely on Nissl bodies as their primary sites of protein synthesis. A decrease or loss of Nissl bodies typically impaired neuronal function and is commonly associated with neuronal degeneration or necrotic [21]. ZEA and its metabolites can cross the blood–brain barrier and accumulate in brain tissue, leading to neurotoxic effects [22]. In this study, neuronal atrophy and dissolution of Nissl bodies in the hypothalamus, particularly the presence of vacuoles and ghost cells in the high-dose group (3.0 mg/kg), provided direct evidence of ZEA-induced neurotoxicity, which suggests that ZEA may cause irreversible neuronal damage or cell death through mechanisms such as oxidative stress and apoptosis [23]. As the central regulator of the neuroendocrine system, the hypothalamus is highly vulnerable to such injury, which may disrupt the synthesis and secretion of neurohormones, including GnRH, thereby impairing pituitary regulation. Collectively, these findings highlight the critical role of hypothalamic integrity in maintaining neuroendocrine homeostasis. Nevertheless, the exact mechanism by which ZEA compromises hypothalamic integrity remains to be fully elucidated and warrants further investigation.

With increasing dose of ZEA the proportion and size of acidophilic cells in the pituitary gland increased, whereas those of basophilic cells decreased. Prolactin cells, a subtype of acidophils, are primarily responsible for prolactin secretion [24]. Estrogen is known to stimulate the proliferation and secretory activity of prolactin cells [25]. Therefore, ZEA acts as a potent estrogen-like fungal toxin, promoting the proliferation and hypertrophy of eosinophils. Supporting this, previous studies have reported that ZEA exposure significantly increases serum prolactin levels in prepubertal gilts [26], which aligns with our observation of elevated acidophilic cell abundance. In conversely, the reduction in basophilic cells, which predominantly secrete FSH and LH, indicates an inhibitory effect of ZEA on the reproductive axis. Zearalenone (ZEA) and its metabolites have been reported to inhibit the synthesis of follicle-stimulating hormone (FSH) in porcine pituitary cells and luteinizing hormone (LH) in bovine anterior pituitary cells, an effect mediated by the non-classical estrogen membrane receptor, GPR30 [27,28]. Such inhibition of gonadotrophs may represent a significant histological basis for ZEA-induced reproductive impairments, including infertility and abortion. Taken together, this study offers critical morphological insights linking ZEA-induced neuroendocrine toxicity to reproductive impairment.

### 3.2. Effects of ZEA on the Expression of GPR30, Kiss1-GPR54-GnRH/GnRHR in Hypothalamus and Pituitary Gland

To elucidate the molecular mechanisms underlying the observed histological alterations, the expression profiles of key reproductive regulatory genes within the hypothalamic–pituitary axis were systematically evaluated following graded ZEA exposure. Our findings demonstrated that ZEA exhibits a non-monotonic, dose-dependent, biphasic regulatory effect on the hypothalamic Kiss1-GPR54-GnRH/GnRHR pathway, characterized by stimulation at low doses and inhibition at high doses. In contrast, hypothalamic GPR30 expression was progressively upregulated with increasing ZEA concentrations. Conversely, ZEA exhibited a direct inhibitory effect on Kiss1-GPR54 signaling pathway in the pituitary gland.

GPR30, a G-protein-coupled estrogen receptor responsible for mediating rapid non-genomic estrogen signaling, is abundantly expressed in the hypothalamus [29]. In the present study, as the dietary ZEA concentration increased, IOD along with mRNA and protein expression levels of GPR30 in both the hypothalamus and pituitary gland rose in a dose-dependent manner, with the highest levels observed at the 3.0 mg/kg dose. Previous research has shown that ZEA significantly upregulates GPR30 expression in anterior pituitary cells of weaned gilts [26], a result highly consistent with our findings. Although studies on ZEA-mediated regulation of hypothalamic GPR30 remain limited, evidence indicates that GPR30 mediates the rapid effects of E2 on GnRH neurons in primates [30]. Consequently, as an estrogen analog, ZEA might upregulate hypothalamic GPR30 expression via a comparable mechanism. Previous laboratory studies have demonstrated that dietary ZEA induces dose-dependent, linear enlargement of the external genitalia and uterus in weaned piglets [31,32]. The external genitalia and uterus are classic estrogen target organs, and their development and weight gain directly reflect elevated estrogenic activity [33]. Collectively, these findings suggest that ZEA may upregulate GPR30 expression in the hypothalamus and pituitary via its estrogenic activity, which in turn elicits estrogen-like responses in peripheral reproductive tissues.

Taken together, these findings suggest that ZEA may contribute to the upregulation of GPR30 expression in the hypothalamus and pituitary gland through its estrogen-mimicking actions, thereby eliciting estrogen-like responses in peripheral reproductive tissues. However, it must be emphasized that GPR30 does not function in isolation. Classical estrogen receptors (ERα and ERβ) are indispensable for mediating xenoestrogenic feedback within the central HPG axis [34]. Although our current investigation focused on elucidating the contribution of GPR30, the profound neuroendocrine disruption observed is highly likely the result of a synergistic interplay between GPR30-driven rapid signaling and classical ER-mediated genomic pathways [35]. Therefore, while GPR30 is a key contributor, future research co-evaluating both receptor systems is essential to fully untangle their respective and concurrent roles in ZEA toxicity.

The hypothalamic Kiss1-GPR54-GnRH/GnRHR system constitutes a central regulatory network governing the reproductive axis. Specifically, Kiss1-GPR54 system directly regulates GnRH-expressing neurons in the brain, which trigger sex steroid production. This system is considered a neuroendocrine switch for the initiation of puberty [36]. Subsequently, GnRH binds to GnRHR in the pituitary gland, stimulating the synthesis and secretion of FSH and LH, which subsequently regulate follicular development, ovulation, spermatogenesis, as well as steroidogenesis [37]. In contrast to the linear upregulation of GPR30, the expressions of Kiss1, GPR54, GnRH, and GnRHR demonstrated a pronounced biphasic response to ZEA. Specifically, their expression levels peaked at 1.5 mg/kg but were sharply downregulated at 3.0 mg/kg, dropping below those of both the control and 0.15 mg/kg groups. This pattern suggests that low doses of ZEA may function as estrogen agonists, activating Kiss1 neurons and stimulating the entire GnRH secretion pathway [38]. This observation aligns with prior evidence suggesting that low-dose ZEA exposure can induce overstimulation symptoms, such as precocious puberty and superovulation in animals [39]. When the ZEA dose was increased to 3.0 mg/kg, the expressions of Kiss1, GnRH, and GnRHR was significantly reduced. This suppression may arise from the interplay of multiple regulatory mechanisms. First, sustained high-intensity estrogenic signaling may trigger classical negative feedback, suppressing Kiss neuronal activity [40]. In addition, prolonged or high-dose exposure to estrogenic agonists may induce desensitization and internalization of GPR54 and GnRHR, ultimately reducing their signal responsiveness [41]. More importantly, the effects of high-dose ZEA may extend beyond classical estrogenic pathways to induce severe, generalized cytotoxicity [42]. Elevated ZEA levels provoke massive oxidative stress, mitochondrial dysfunction, and endoplasmic reticulum stress, which rapidly trigger apoptotic cascades in neural tissues [23,43]. Therefore, the extensive downregulation of the Kiss1/GPR54/GnRH axis and overt histological damage observed at 3.0 mg/kg are likely driven by a dual mechanism: intense endocrine negative feedback coupled with direct, non-estrogenic neurotoxicity caused by heavy ZEA accumulation. Consistent with our findings, previous studies have revealed pronounced downregulation of GnRHR expression in the brains of rats exposed to 20 mg/kg ZEA [44]. In summary, this study uncovered a distinct non-linear, biphasic regulatory pattern of ZEA within the hypothalamic–pituitary–gonadal axis in weaned gilts.

The Kiss1–GPR54 system contributes to the release of FSH and LH in response to GnRH stimulation in pituitary gland. Furthermore, in vitro research using porcine pituitary cells further indicates that Kiss1 and GPR54 play a pivotal role in modulating gonadotropin secretion [45]. This study demonstrated that pituitary expression of Kiss1 and GPR54 was significantly downregulated following exposure to moderate and high doses (1.5 and 3.0 mg/kg) of ZEA. Kiss1 regulates gonadotropin secretion, along with other pituitary hormones, such as growth hormone and prolactin, through endocrine, paracrine, and autocrine pathways [46]. Consistent with this, earlier laboratory studies have reported that diets containing 3.0 mg/kg ZEA markedly reduces serum levels of FSH and LH [47]. Except under 3.0 mg/kg ZEA exposure, pituitary GnRH levels remained relatively stable across most treatments. This pattern might arise because the pituitary gland typically receives a relatively stable input of GnRH and only experiences an increase in GnRH to restore homeostasis when hypothalamic secretion decreases. Given the limited understanding of pituitary Kiss1–GPR54 signaling and the scarcity of available evidence, other pathways may also contribute to the effects of ZEA on pituitary Kiss1–GPR54 expression and subsequent gonadal function. In this experiment, pituitary GnRHR level increased at lower ZEA doses but declined with higher exposure levels. These observations align with previous reports showing that central administration of endorphins or naloxone, as well as peripheral lipopolysaccharide injection, inducing coordinated alterations in hypothalamic GnRH secretion and pituitary GnRHR expression in ewes [48,49]. This similarity suggests that high levels of ZEA exposure may trigger hypothalamic responses analogous to those in earlier studies. However, further experimental evidence is required to confirm these conclusions.

While our findings reveal the complex, non-linear effects of ZEA on the central Kiss1/GPR54 system, the study is not without limitations. Specifically, using whole hypothalamic tissues masks nucleus-specific responses, lacking ERα/ERβ data restricts understanding of receptor interplay, and high-dose neurotoxicity complicates distinguishing endocrine disruption from general cytotoxicity, highlighting the need for future chronic, low-dose research.

## 4. Conclusions

In conclusion, ZEA disrupts the central neuroendocrine system of weaned gilts through a dose-dependent, biphasic modulation of the Kiss1/GPR54/GnRH axis. While moderate ZEA exposure stimulates this pathway via estrogenic agonism, high doses induce severe suppression driven by overwhelming negative feedback and neurotoxicity. Concurrently, the persistent upregulation of GPR30 highlight its potential role as a key player in mediating ZEA’s persistent estrogenic signaling. Ultimately, ZEA-induced reproductive toxicity is a highly non-linear process, driven by an intricate interplay between specific endocrine interference and generalized tissue damage across the hypothalamus and pituitary.

## 5. Materials and Methods

### 5.1. Preparation of Zearalenone Diet

Purified ZEA was obtained from Fermentek (Jerusalem, Israel). Purified ZEA dissolved in ethyl acetate was blended with mycotoxin-free corn meal and evaporated overnight to yield a 1000 mg/kg primary stock. This stock was diluted with mycotoxin-free corn meal to a 10 mg/kg working premix, which was then incorporated into the basal diet to achieve target ZEA concentrations of 0.15, 1.5, and 3.0 mg/kg. The control diet received an identical vehicle-treated premix. All diets were prepared in a single batch and stored at 4 °C in sealed containers [19].

### 5.2. Animals, Treatments and Feeding Management

All procedures involving the rearing of weaned gilts adhered to the Chinese Ministry of Agriculture’s ethical standards and received approval from the Animal Nutrition Research Institute at Shandong Agricultural University. For this study, 32 healthy weaned gilts (Duroc × Landrace × Yorkshire, 35 days old), weighing an average of 12.39 ± 0.24 kg, were partitioned into four dietary groups, with eight females per treatment assigned to a basal diet (Table 1) contained 0 (Control), 0.15 (ZEA0.15), 1.5 (ZEA1.5) and 3.0 (ZEA3.0) mg/kg ZEA. The basal diet was designed to meet or exceed the nutrient requirements recommended by NRC (2012) [50]. The analyzed ZEA concentrations in the diets were 0, 0.14 ± 0.00, 1.45 ± 0.02, and 3.12 ± 0.07 mg/kg, respectively. During the 32-day experimental at the Animal Research Station of Shandong Agricultural University (Taian, China), each gilt was confined to a separate stainless-steel metabolic crate (0.48 m^2^). These pens featured plastic slatted floors along with individual feeders and nipple drinkers. Ambient temperature was initially controlled at 30 °C for the first 7 days, and subsequently maintained at 26–28 °C, with relative humidity kept at approximately 65%.

### 5.3. Sample Collection

Following a 12-h fast on day 32, all gilts were euthanized for tissue collection. The sampled hypothalami and pituitary glands were allocated for either molecular or histological evaluations. For the analysis of protein and gene expression, one hypothalamic hemisphere per animal and four pituitaries per group were cryopreserved in liquid nitrogen before long-term storage at −80 °C. The remaining hypothalamic hemispheres and pituitaries were fixed in Bouin’s fluid prior to paraffin embedding for H&E staining and immunohistochemistry (IHC).

### 5.4. Histological Examination

Hypothalamic (8 μm) and pituitary (6 μm) sections were prepared utilizing a Leica RM 2235 microtome (Leica Microsystems, Wetzlar, Germany). Following deparaffinization and rehydration through graded ethanol series (from 100% down to 70%), standard H&E staining was performed. Briefly, sections were incubated in hematoxylin for 2 min, differentiated with 1% acid-alcohol, blued, and counterstained with eosin for 2 min. Post staining, the slides were dehydrated, coverslipped, and examined under a Nikon Eclipse 80i microscope (Tokyo, Japan).

### 5.5. Immunohistochemistry

Immunohistochemical analysis was conducted based on the protocol described by Yang et al. [19]. Briefly, following deparaffinization and rehydration, tissue sections underwent heat-induced antigen retrieval via microwave irradiation (20 min) in 0.01 M sodium citrate buffer (pH 6.0). To minimize background noise, endogenous peroxidases were deactivated by 10% H_2_O_2_ (40 min), followed by a 1.5-h application of 10% goat serum (ZSGB-BIO). Sections underwent overnight incubation at 4 °C with a panel of rabbit primary antibodies, as listed below: anti-Kiss1 (1:150, bs-0749R), anti-GPR54 (1:150, bs-2501R), anti-GPR30 (1:100, bs-1380R) (all from BIOSS, Beijing, China), as well as anti-GnRH (1:200, 26950-1-AP) and anti-GnRHR (1:200, 19950-1-AP) (both from Proteintech, Beijing, China). Subsequent to PBS washes, slides were treated with a Polink-2 Plus Polymer HRP anti-rabbit detection system for 1 h at 37 °C. Immunoreactivity was visualized using a DAB chromogen kit (TIANGEN PA110, Beijing, China) for 1–3 min. Finally, sections were dehydrated, cleared, mounted, and examined microscopically. Negative controls, omitting the primary antibodies, were included concurrently to confirm staining specificity.

### 5.6. Integrated Optical Density Measurement

The immunoreactivity and spatial distribution of GPR30, Kiss1, GPR54, GnRH, and GnRHR were evaluated utilizing a Nikon ECLIPSE 80i microscope (Tokyo, Japan) according to Yang et al. [19]. For each sample, three stained sections were randomly selected, and three non-overlapping visual fields per section were specifically selected from the arcuate nucleus (ARC) region for observation and imaging. The total cross-sectional integrated optical density (IOD) of the acquired images was quantified utilizing Image-Pro Plus 6.0 software (Media Cybernetics).

### 5.7. Total RNA Extraction and Real-Time Quantitative RT-PCR (qRT-PCR)

Following the protocol detailed by Wan et al. [31], total RNA was extracted with RNAex Pro Reagent (Accurate Biology, Changsha, China). After verifying RNA yield and quality, cDNA synthesis was performed via the PrimeScript RT Master Mix (TaKaRa Bio, Shiga, Japan, RR036A). Transcript abundances of Kiss1, GPR54, GnRH, GnRHR, and GPR30 were subsequently quantified utilizing the SYBR Green Premix Pro Taq HS qPCR Kit (Accurate Biology, AG11701). Triplicate measurements were performed for each sample, and the 2-ΔΔCT method was applied to determine relative gene expression levels. The utilized primer sequences and corresponding amplification lengths are listed in Table 2.

### 5.8. Western Blotting

Following the protocol described by Yang et al. [32], total protein was extracted using RIPA lysis buffer supplemented with PMSF (Beyotime Biotechnology, Shanghai, China). Protein concentrations were determined via a BCA assay kit (Beyotime), and all samples were normalized to a final loading amount of 25 μg. Proteins were resolved by SDS-PAGE and electrotransferred onto nitrocellulose membranes. After blocking with 5% non-fat milk, the membranes were probed overnight at 4 °C with the following primary antibodies: anti-Kiss1 (bs-0749R), anti-GPR54 (bs-2501R), and anti-GPR30 (bs-1380R) (all diluted 1:500; BIOSS, Beijing, China), as well as anti-GnRH (26950-1-AP) and anti-GnRHR (19950-1-AP) (both diluted 1:500; Proteintech, Beijing, China). Following three washes in TBST, the blots were incubated with an HRP-conjugated anti-rabbit secondary antibody (1:1000, Beyotime) for 1 h. Immunoreactive bands were visualized using the BeyoECL Plus reagent (Beyotime) and captured on a FusionCapt Advance FX7 imaging system (Beijing Oriental Science and Technology Development Co., Ltd., Beijing, China). Band intensities were densitometrically quantified utilizing Image-Pro Plus 6.0 software (Media Cybernetics, Silver Spring, MD, USA).

### 5.9. Statistical Analysis

All quantitative data were first normalized to ensure comparability across samples. These normalized data were then evaluated for the assumptions of ANOVA. Specifically, the data were tested for normality using the Shapiro–Wilk test with *p* > 0.05 considered to follow a normal distribution and for homogeneity of variance using Levene’s test. For variables that failed to meet these assumptions, log10 transformation was applied to the normalized data to achieve normality and homoscedasticity. The processed data were analyzed using a one-way ANOVA model within SAS 9.2 software (SAS Institute Inc., Cary, NC, USA). The specific linear and quadratic responses to graded concentrations of ZEA were evaluated through orthogonal polynomial contrasts. Multiple comparisons were conducted with Tukey’s post hoc test, and significance was declared at *p* < 0.05.

## Figures and Tables

**Table 1:** Ingredients and nutrient contents of the basal diet (air-dry basis) %.

Ingredients	Content	Nutrients ^2^	Content
Expanded corn	64.43	Metabolizable energy, MJ/kg	13.86
Whey powder, CP 3%	5.00	Crude protein	18.48
Fermented soybean meal	14.00	Calcium	0.74
Expanded soybean	8.50	Total phosphorus	0.62
Fish meal	4.00	STTD phosphorus	0.41
CaHPO4	1.15	ATTD phosphorus	0.38
PulverizedLimestone	0.70	Available phosphorus	0.39
NaCl	0.20	Lysine	1.38
L-Lysine HCl	0.76	Methionine	0.40
DL-Methionine	0.08	Sulfur amino acid	0.66
L-Threonine	0.16	Threonine	0.85
L-Tryptophan	0.02	Tryptophan	0.23
Premix ^1^	1.00		
Total	100.00		

^1^ Supplied per kilogram of diet: vitamin A, 2300 IU; vitamin D_3_, 230 IU; vitamin E, 20 IU; vitamin K_3_, 0.60 mg; vitamin B_1_, 1.80 mg; vitamin B_2_, 4.25 mg; vitamin B_6_, 2.00 mg; vitamin B_12_, 0.020 mg; Pantothenic acid, 13.00 mg; Niacin, 20.00 mg; Biotin, 0.09 mg; Folic acid, 0.45 mg; Mn (Manganese methionine), 4.00 mg; Fe (Iron (II) fumarate), 90 mg; Zn (Glycine zinc), 90 mg; Cu (bis(glycinato)copper), 6.00 mg; I (Calcium Iodate), 0.14 mg; Se (Selenium Yeast), 0.30 mg. STTD, standardized total tract digestible; ATTD, apparent total tract digestible. ^2^ The value for Metabolizable energy was calculated; all other nutrient levels were determined by analysis.

**Table 2:** Primers sequences of Kisspeptin-1 (Kiss1), G-protein-coupled receptor 54 (GPR54), Gonadotropin-releasing hormone (GnRH), Gonadotropin-releasing hormone receptor (GnRHR), G-protein-coupled receptor 30 (GPR30).

Target Gene	Accession No.	Primer Sequence (5′ to 3′)	Product Size, bp
KISS1	NM_001134964.1	F: GTCCGCCTACAACTGGAACTC	105
		R: GTTTGAAGGTCTCAGTCTCGC	
GPR54	NM_001044624.2	F: CTCAACTACATCCAGCAGGTCTC	93
		R: CAGCGGAAACACAGTCACATAC	
GnRH	NM_214274.1	F: GCTCGACTAGCAGAACCTCAG	108
		R: CCCATTTCCTCTTCAATCAGACT	
GnRHR	NM_214273.1	F: TGCCTCTTCATTATCCCTCTCCTC	131
		R: GCTCGTGGTATGTTGTTCTTGGA	
GPR30	XM_003124244.5	F: GAGCACCAGCAGTACGTCATC	102
		R: GTTCACCACCAGGATCAGGAG	
β-actin	XM_021086047.1	F: GGACTTCGAGCAGGAGATGG	138
		R: AGGAAGGAGGGCTGGAAGAG	

## Data Availability

The original contributions presented in this study are included in the article. Further inquiries can be directed to the corresponding authors.

## Abbreviations

The following abbreviations are used in this manuscript:

ZEA	Zearalenone
α-ZOL	Alpha-zearalenone
HPG	Hypothalamic-pituitary-gonadal
GnRH	Gonadotropin-releasing hormone
FSH	Follicle-stimulating hormone
LH	Luteinizing hormone
Kiss	Kisspeptin
KP	Kisspeptin
BW	Body weight
BCA	Bicinchoninic acid
